# Dermatological Manifestations in Patients With Chronic Kidney Disease: A Review

**DOI:** 10.7759/cureus.52253

**Published:** 2024-01-14

**Authors:** David Arriaga Escamilla, Alisha Lakhani, Sneha Antony, Karla N Salazar Villegas, Manasvi Gupta, Parameswaran Ramnath, María Isabel Murillo Pineda, Alexandra Bedor, Douglas Banegas, Ernesto Calderon Martinez

**Affiliations:** 1 Internal Medicine, Universidad Justo Sierra, Mexico City, MEX; 2 Medicine, Research MD, Vadodara, IND; 3 Medicine, Shantabaa Medical College, Amreli, IND; 4 Pharmacology, K S Hegde Medical Academy, Mangalore, IND; 5 Dermatology, Hospital General de Zona 29, Ciudad de Mexico, MEX; 6 General Practice, Jawaharlal Nehru Medical College, Aligarh, IND; 7 Medicine, Jonelta Foundation School of Medicine, Manila, PHL; 8 Primary Care, Universidad Católica de Honduras, Tegucigalpa, HND; 9 Internal Medicine, Instituto Salvadoreño del Seguro Social, San Salvador, SLV; 10 General Medicine, Universidad Nacional Autonoma de Honduras, San Pedro Sula, HND; 11 Digital Health, Universidad Nacional Autónoma de México, Ciudad de Mexico, MEX

**Keywords:** chronic kidney disease, xerosis, pruritus, nephrogenic systemic fibrosis, caciphylaxis, access site infections, pseudoporphyria, porphyria cutanea tarda, acquired perforating dermatoses, dermatoses

## Abstract

Chronic kidney disease (CKD) is a progressive disease and has multiple clinical manifestations; when CKD reaches the end stage, at least one cutaneous manifestation appears due to some increased toxin levels or a constant proinflammatory state. Nonspecific manifestations include pruritus, xerosis, pigmentation disorders, acquired ichthyosis, purpuric spots, and nail disorders. Some specific manifestations are bullous dermatoses, acquired perforating dermatoses (APD), eruptive xanthoma, access site infections, calcifying disorders, and nephrogenic systemic fibrosis (NSF). All these cutaneous changes negatively impact patients; early recognition and diagnosis of these dermatoses will make a difference in their quality of treatment. Exploring a patient's skin is fundamental to suspect some diseases and increased toxin levels; pruritus occurs when uremic toxins are raised, and nail disorders are associated with hypoalbuminemia. This review provides the clinician with information on the clinical manifestations that occur in CKD, including epidemiology, pathophysiology, clinical manifestations, diagnosis, histopathology, treatment, and life impact of the dermatoses in CKD.

## Introduction and background

Chronic kidney disease (CKD) is a progressive, non-communicable disease affecting more than 800 million individuals globally. With increasing risk factors like hypertension, diabetes mellitus, and obesity, there has been a significant rise in CKD incidence [1]. It has become one of the leading causes of death in the 21st century, according to the Global Burden of Disease [2,3], and is more prevalent in low- and middle-income countries than in high-income nations [4]. The Centers for Disease Control and Prevention's CKD Surveillance System reports that approximately one in seven adults in the United States is diagnosed with CKD stages 1-4. From 2017 to March 2020, the crude prevalence of CKD increased to 14.8% [5]. CKD is a progressive loss of kidney function or an estimated glomerular filtration rate (eGFR) of less than 60 mL/min/1.73 m², persisting for three months or more, regardless of the cause [6]. CKD is diagnosed through laboratory tests measuring glomerular filtration rate (GFR) or by detecting the presence of albumin, protein, or both [4]. The etiology of CKD varies globally; the most common primary causes are type 2 diabetes mellitus (30%-50%) and hypertension (27.2%) [7].

CKD manifests in various ways, and 50%-100% of patients with end-stage renal disease (ESRD) exhibit at least one associated cutaneous change, ranging from asymptomatic to life-threatening symptoms. These can be divided into specific and nonspecific forms [8]. The nonspecific manifestations include generalized pruritus, xerosis, pigmentation disorders, acquired ichthyosis, purpuric spots, and Lindsay’s nails [9]. Pruritus is the most common nonspecific symptom in patients with ESRD, affecting around 90% of those on hemodialysis (HD), significantly impacting their quality of life. The presence of pruritus is linked to a poor prognosis. Manifestations include bullous dermatoses, acquired perforating disorders, eruptive xanthoma, calcifying disorders, and nephrogenic systemic fibrosis (NSF). Among the specific manifestations, only NSF is considered unique to CKD [9].

## Review

Acquired perforating disease of HD 

In a range of conditions known as acquired perforating dermatoses (APD), material from the dermis is expelled transepidermally with minimal harm to adjacent structures. APD commonly occurs in patients with diabetes or ESRD [10] and can affect up to 11% of HD patients. APD typically develops in those with diabetes or chronic renal failure [11,12]. The exact cause of APD is unknown. It is hypothesized that dermal microdeposits of substances like calcium salts, possibly resulting from chronic renal failure, may trigger the condition [12]. In diabetic individuals, microangiopathy is also identified as a key predisposing factor for APD, particularly in those who scratch their itchy skin. Scratching-induced damage could lead to dermal necrosis due to impaired blood flow from vasculopathy, with the necrotic dermal material eventually protruding through the epidermis [13,14].

**Figure 1:**
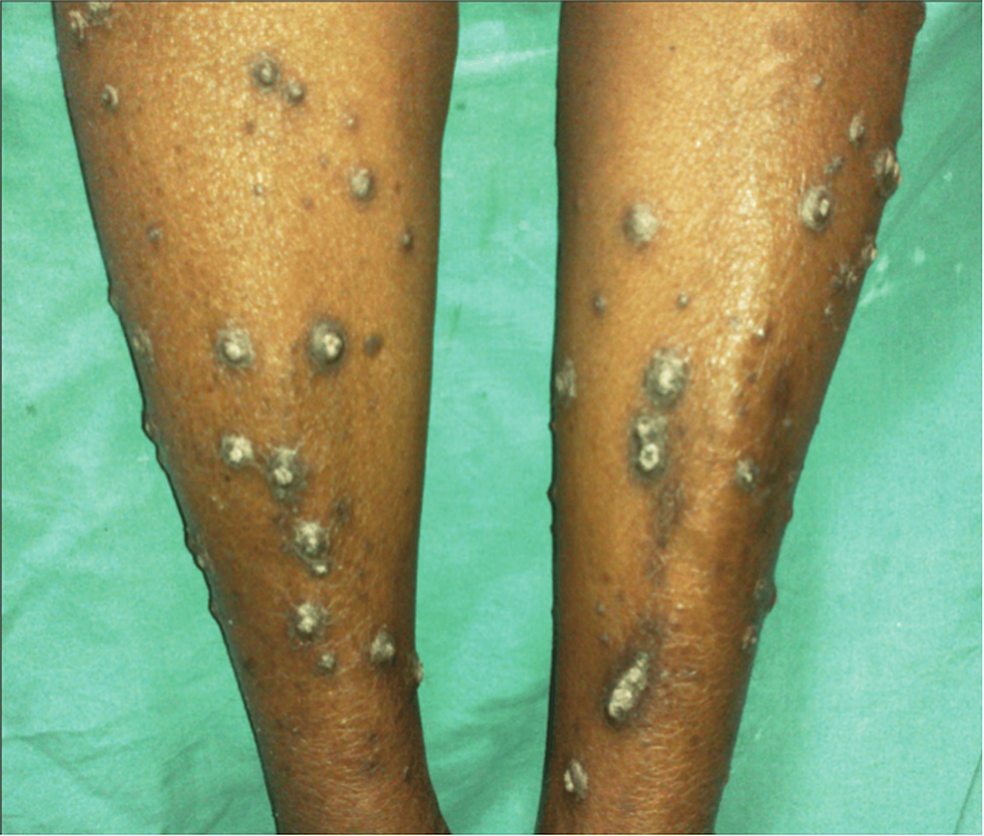
Acquired perforating disease of hemodialysis Affection of bilateral shins.

APD can be diagnosed via skin biopsy [15]. Differential diagnosis is crucial as several conditions can mimic APD clinically, including prurigo nodularis, folliculitis, arthropod bites, multiple keratoacanthomas, psoriasis, and lichen planus. These should be ruled out through histopathology [16,17]. Typically, APD presents with dispersed, widespread pruritic papules and nodules featuring central horny plugs on the trunk and extremities, with the head being a less common site [14]. Histopathology reveals a keratotic plug composed of keratin, collagen, or elastic fibers, along with neutrophils in an epidermal invagination or dilated hair follicle [14]. The most effective treatment for APD often involves the concurrent use of retinoids and potent topical steroids. Management also includes medications to alleviate itching, such as sedative antihistamines [18]. Systemic therapy may include retinoids, glucocorticoids, and phototherapy. Narrow-band ultraviolet B phototherapy is recommended for its effectiveness in reducing concurrent uremic pruritus [19]. Figure 1 represents a clear example of this condition [20].

Access site infections

HD and peritoneal dialysis (PD) are common techniques for filtering the urea and toxin load in patients with CKD. Common access sites for HD include a central venous catheter (CVC), arteriovenous fistulae like the Cimino-Brescia fistula, and vascular grafting [21]. CVCs, which come in tunneled and non-tunneled varieties, have a higher infection rate in the former [22]. Prompt identification and proper management of these infections are critical to avoid complications such as lumen thrombosis with blockage, stenosis, infection, and biofilm formation, which increase morbidity and prolong hospital stays [23].

Infections are commonly caused by gram-positive cocci like Staphylococcus aureus and coagulase-negative staphylococci (CONS). Gram-negative organisms like E. coli, Klebsiella pneumoniae, polymicrobial infections, or fungal infections in immunocompromised patients may also be present [21]. Blood cultures or catheter tip cultures are typically conclusive. S. aureus and CONS can produce extracellular mucopolysaccharides, forming a “biofilm” that helps the organisms evade the immune system and reduce antibiotic penetration [21]. Increased susceptibility to infection may be linked to factors like an immunosuppressed state, history of bacteremia, poor hygiene practices, adjacent skin infection, high BMI, iron overload states, and hypoalbuminemia [22,23].

Systemic clinical signs include fever or hypothermia, chills, malaise with easy fatigability, nausea or vomiting, and altered mental sensorium. These may progress to septic shock (hemodynamic instability), leading to organ dysfunction and potentially death [22,23].

Infection prevention in dialysis patients begins with educating patients and staff about standard aseptic protocols and hygiene maintenance, including hand-washing etiquette and the use of gloves and masks during catheter insertion or manipulation. The site should be cleaned with an antiseptic, preferably chlorhexidine >0.5% with alcohol, 70% alcohol, or 10% povidone-iodine solution, during catheter insertion [24].

Local infections should be treated with appropriate antibiotics after confirming the organism and its susceptibility. Symptomatic treatment includes analgesics for pain control, antipyretics, and multivitamin supplements. For disseminated infections, maintaining hemodynamic status, utilizing blood or catheter-tip culture for organism identification and antibiotic sensitivity, and administering appropriate medications are crucial [23,25].

Calcifying disorders 

Vascular calcification is a well-established risk factor for mortality in patients with kidney disease. Calciphylaxis, where calcification involves large and medium-sized arteries, is primarily linked to longstanding hyperphosphatemia and pseudohypoparathyroidism [26-28]. Calciphylaxis is characterized by calcification of arterioles and capillaries in the dermis and subcutaneous adipose tissue. Commonly affected areas include the fatty regions of the thighs, abdomen, buttocks, and lower extremities. Lesions, firm with a pink or mottled color or livedo reticularis-like appearance, progress to painful ulcers with black eschar. Surrounding skin may exhibit mottling and reticulate dyspigmentation. Radiologic investigations can reveal linear calcium deposits in the skin, and histopathology typically shows calcification of medium-sized vessels with intimal hyperplasia and thrombosis [27,29].

Prevention and reversal of calcium-phosphate precipitation involve the administration of sodium thiosulfate. Treatment also includes correcting calcium, phosphorus, and parathyroid hormone levels using non-calcium-containing phosphate binders, such as sevelamer carbonate or lanthanum carbonate. Cinacalcet may be used to suppress PTH levels. Medications contributing to calciphylaxis, including vitamin D, calcium supplements, and warfarin, must be withheld [30].

Calcinosis cutis, characterized by benign nodular calcification due to metastatic deposition of calcium salts in the skin and subcutaneous tissues, is often associated with elevated calcium-phosphate products. Hyperparathyroidism may be present. Lesions appear as firm white papules, plaques, and nodules, usually over periarticular areas and fingertips. Histopathology reveals irregular deposits of intensely basophilic acellular material in the dermis and subcutaneous tissue, typically well-circumscribed, with a thin rim of eosinophilic hyalinization and often a host giant cell reaction [31,32]. Treatment focuses on normalizing calcium and phosphate levels, and if hyperparathyroidism is present, parathyroidectomy may be beneficial. Phosphate binders and dietary phosphate reduction are advised. Other treatments include vinpocetine, sodium thiosulfate, and intravenous pamidronate, with surgical removal considered for symptomatic lesions [33].

**Figure 2:**
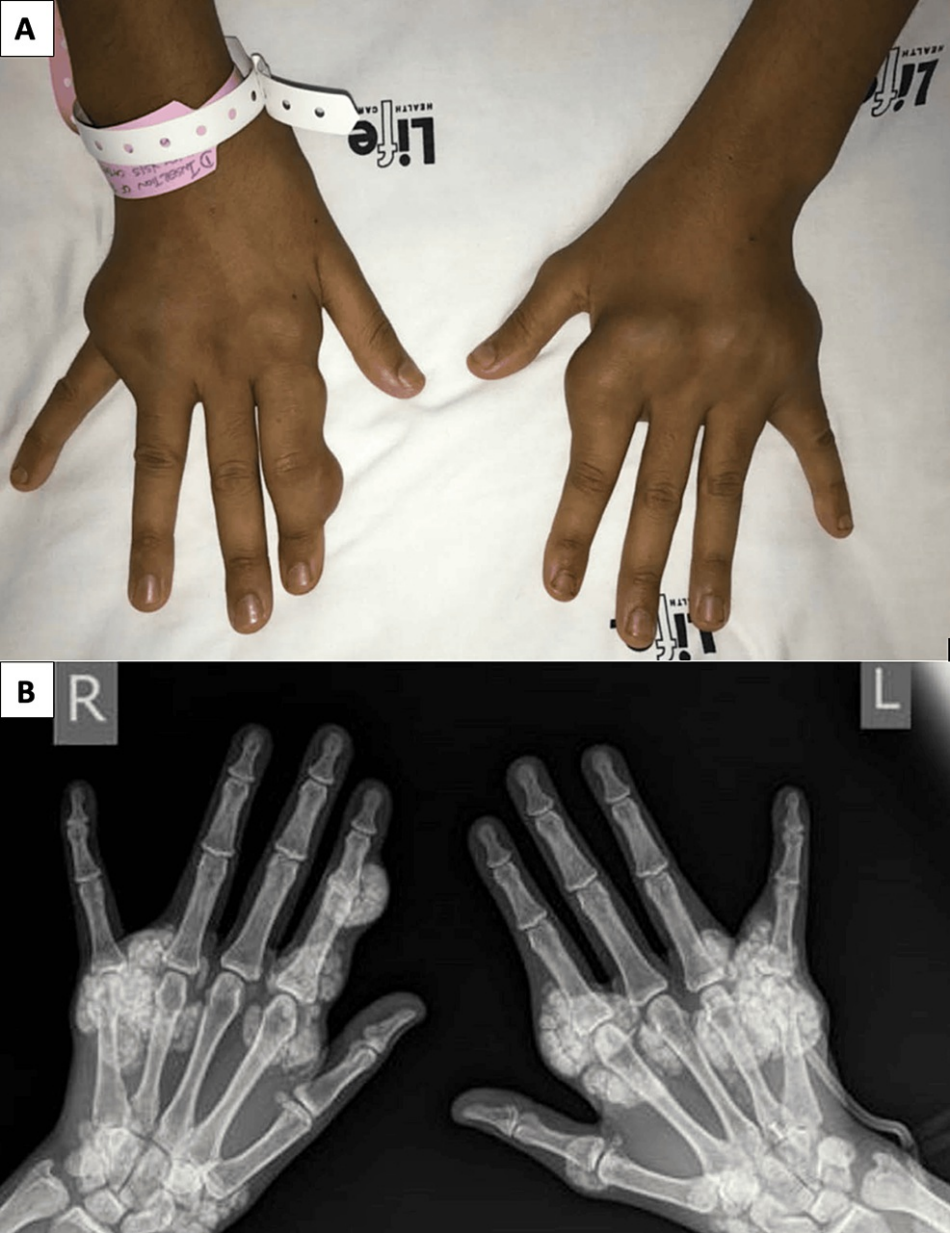
Metastatic calcinocis cutis (A) Nodules predominantly affecting the metacarpophalangeal joints of both hands. (B) Plain radiograph showing periarticular carpal, metacarpophalangeal joints, and subcutaneous calcified nodules without bony erosions.

Metastatic calcification in ESRD patients can cause a range of pathologies, including extraosseous soft tissue and solid organ calcification, and lung symptoms like dyspnea and chronic, nonproductive cough [34-38]. Treatment includes calcium-free phosphate binding agents, bisphosphonate therapy, treatment for aluminum overload, or successful renal transplantation for marked clinical improvement [34,39]. An example of metastatic calcinosis cutis as nodules in hands is presented in Figures 2A, 2B [40].

Bullous dermatosis: porphyria cutanea tarda (PCT) and pseudoporphyria

**Figure 3:**
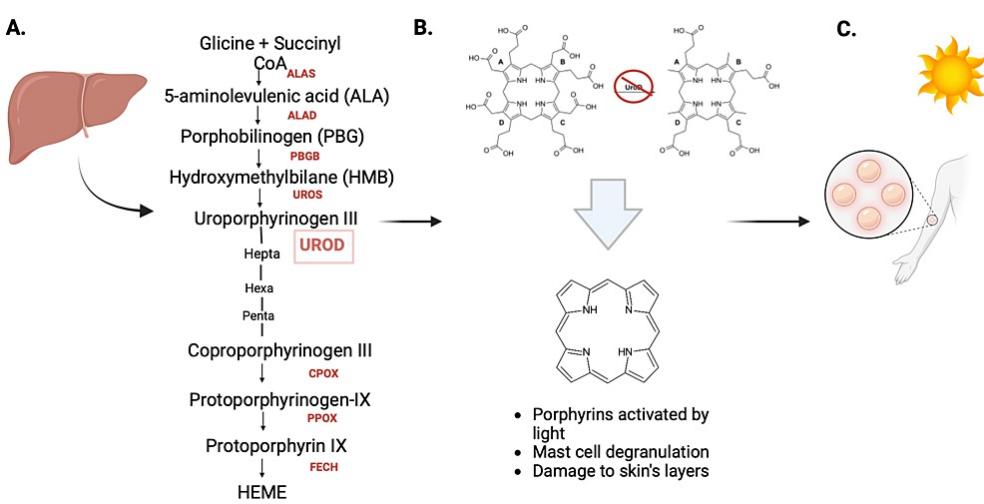
Porphyria cutanea tarda (A) Scheme of the heme biosynthesis in the liver accomplished by the sequential action of eight enzymes. (B) When hepatic UROD is inhibited, uroporphyrinogen I, III, and the reaction's partially decarboxylated intermediates (i.e., hepta-, hexa-, and penta-carboxylporphyrinogen) build up in the liver and eventually travel from hepatocytes to plasma where they auto-oxidize to the corresponding porphyrins. (C) The accumulation of the porphyrins is typically chronic, causing persistent cutaneous photosensitivity or skin fragility as a consequence of prolonged exposure to the sun. ALAS - ALA Synthase, ALAD - ALA dehydrase, PBG deaminase, UROS - Uropotphyrinogeno III Synthase, UROD - Uroporphyrinogen Decarboxylase, CPOX - Coproporphyrinogen oxidase, PPOX - Protoporphyrinogen oxidase, FECH - Ferrochelatase [41,42]. Created by: Alexandra Bedor on www.biorender.com

Porphyrias are primarily inherited disorders of heme biosynthesis, leading to acute neurovisceral attacks or cutaneous photosensitivity. PCT, the most prevalent form of cutaneous porphyria, is caused by the inhibition of uroporphyrinogen decarboxylase (UROD). It typically affects adults, more often males, with an estimated prevalence of around 40 per 1,000,000 people [41]. PCT begins when hepatic UROD activity falls to 20% of normal, leading to the accumulation of uroporphyrinogen I, III, and partially decarboxylated intermediates in the liver (Figures 3A-3C). These porphyrins, activated by light in the skin's microcapillaries, cause oxidative damage, leading to skin fragility and blistering [42].

Dermatological findings include chronic bullous lesions on sun-exposed areas like the hands, arms, feet, and face. Diagnosis involves evaluating porphyrins in plasma or urine, with skin biopsy showing subepidermal blistering and other characteristic histological changes [42]. First-line treatments include photoprotection, phlebotomy, and low-dose 4-aminoquinolines [43].

Pseudoporphyria, resembling PCT clinically and histologically but lacking biochemical porphyrin abnormalities, often affects adult women and is associated with NSAID use or tanning bed exposure. Clinical features include vesicles, bullae, and skin fragility on sun-exposed skin, with histologic and immunofluorescent features similar to PCT. Diagnosis requires a thorough medical history, routine histopathological examination, DIF and indirect immunofluorescence, and laboratory evaluation of porphyrins. Treatment involves discontinuing suspected substances and using sun protection, with certain NSAIDs being preferred due to lower photosensitization risk [44].

Nephrogenic systemic fibrosis

NSF is an acquired, idiopathic, chronic, and progressive disease where multiple organs, including the skin (known as Nephrogenic Fibrosing Dermopathy), suffer dysfunction due to pathological pathways leading to fibrosis [45]. It has been associated with the use of Gadolinium for Magnetic Resonance Imaging in CKD patients [46]. Studies have shown that Gadolinium exposure increases levels of transforming growth factor-beta (TGF-β) and interleukin 6 (IL 6) [47-49].

**Figure 4:**
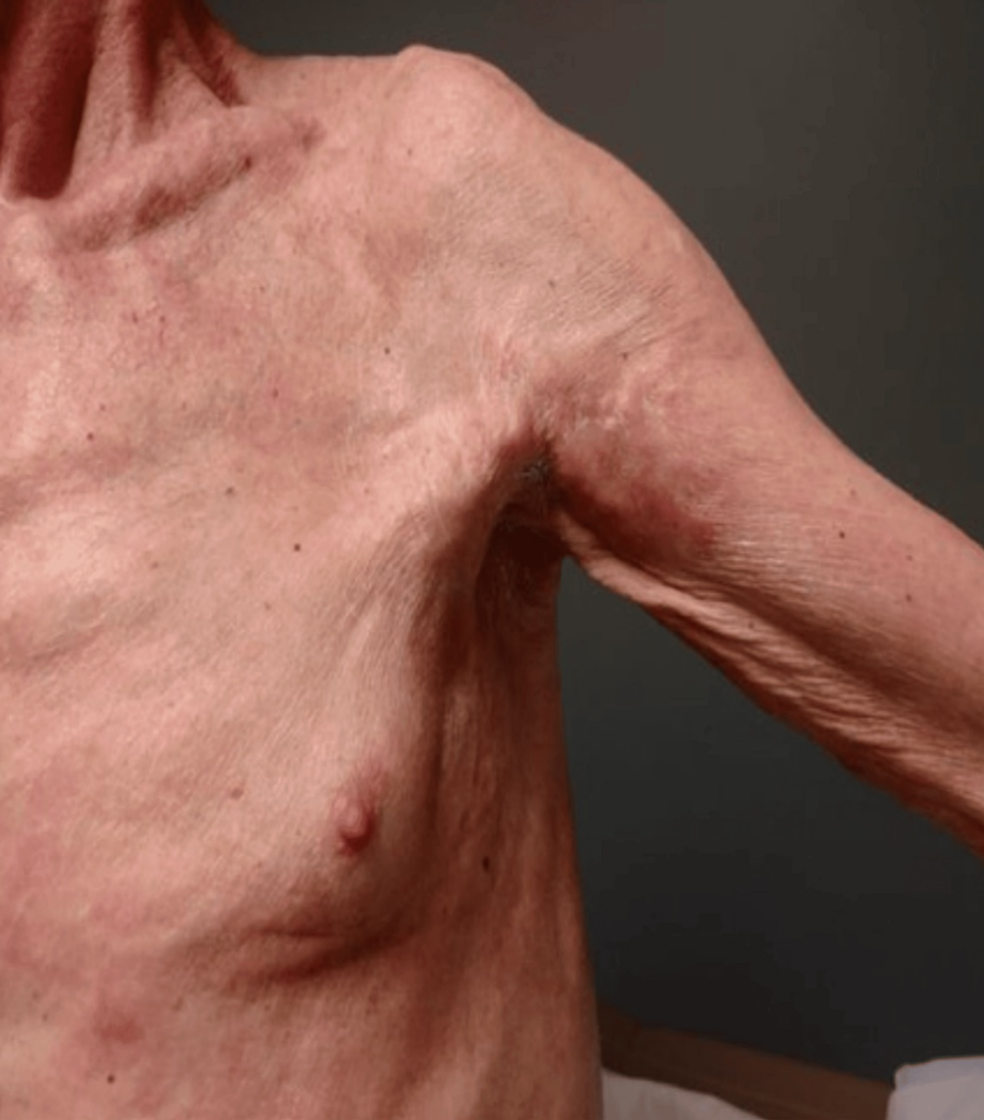
Nephrogenic systemic fibrosis Left upper extremity showing skin thickening and calcified plaque in the axilla.

Within two months of Gadolinium exposure, 90% of patients experience skin color changes to reddish, bluish, or brownish hues. Swelling, often warm, firm, and painful or itchy, occurs in 80% of patients [50]. The most common sites of these dermatoses are the lower extremity (85%), upper extremity (66%), trunk (35%), hands (34%), feet (24%), buttocks (9%), and face (3%) [45]. Up to 50% of patients may experience diffuse hair loss, and some report gastrointestinal symptoms like cramps, vomiting, diarrhea, dyspnea, lymphopenia, anemia, and thrombocytosis or thrombocytopenia (Figure 4) [51,52].

Late symptoms, appearing six to 12 months post-exposure, include skin that swells and evolves into thickened, hardened areas with a peau d’orange appearance. Shiny, atrophic, hairless skin is common, resembling sclerotic skin. Joint motion can be significantly reduced due to contractures, particularly in ankles and knees (55%), leading to severe disability and potential wheelchair dependence [53].

Histopathologically, NSF is characterized by dermal fibrosis with altered collagen bundles and increased dermal spindle cells or fibrocytes [54,55]. Currently, there is no curative treatment, but intensive physiotherapy may help maintain physical abilities and reverse some disabilities [54,55]. Proposed treatments include renal transplant, extracorporeal photopheresis, UV therapy, sodium thiosulfate, corticosteroids, imatinib mesylate therapies, dialysis, and immunosuppressants [56,57]. While HD may partially improve symptoms, renal transplantation has shown more significant improvements, including complete resolution of NSF in some cases [58,59].

Renal pruritus, xerosis, and uremic frost 

CKD-associated pruritus (CKDaP) is a term for various dermatoses characterized by itching in CKD or HD patients. This condition, which can vary in severity, may lead to sleep disorders, anxiety, depression, and restless leg syndrome, and significantly reduce patients' quality of life, causing substantial morbidity [60]. The prevalence of CKDaP ranges from 20% to 90%, with improvement often observed in patients receiving better dialysis care [60,61]. A detailed overview of conditions leading to CKDaP, along with common clinical presentations and potential treatment options, are presented in Table 1 [62-66].

**Table 1:** Conditions that can lead to CKDaP CKD - Chronic Kidney Disease, CKD-aP - Chronic kidney disease associated pruritus, PTH - Parathyroid Hormone, G3a - Stage 3a of CKD, G5d - Stage 5d of CKD

Etiology	Mechanism	Clinical Features	Treatment
Uremic Toxins	Increase of urea leads to its deposition in the dermis and excretion via sweat, causing pruritus [62].	CKD-aP is itching directly related to kidney disease, its severity and distribution may vary from hardly appreciable, to incessant and disturbing [63].	Treatments can be divided into histamine receptor antagonists and mast cell stabilizers [63]
Dermal Xerosis and Uremic Frost	Causing a disruption of dermal sweat and sebaceous glands, an alteration in skin pH, a reduction in skin lipids, and an overall decrease in skin moisture content [64].	Xerosis manifests as dry, scaly skin [63].	Emollients are mainly used to treat xerosis. There are no randomized control trials to suggest which emollient is best [63].
CKD-associated Mineral Bone Disease	High levels of bone turnover, cause pseudohypoparathyroidism and elevated levels of serum phosphate, which combines with calcium, leading to the deposition of calcium phosphate salts in the skin dermis. This irritates the dermal nerve endings, leading to abnormal firing of neurons and itching [65].	Manifests as one or a combination of the following: 1. Calcium, phosphorus, PTH, or vitamin D metabolism. 2. Bone mineralization, volume, linear growth, or strength. 3. Vascular or other soft tissue calcification [66]	Prevention of hyperphosphatemia in patients with CKD stage G3a to G5D may be more important than treatment or normalization of phosphate levels; it includes dietary restriction of phosphate, use of phosphate lowering agents, and dialysis for patients with CKD stage G5D [66].

**Figure 5:**
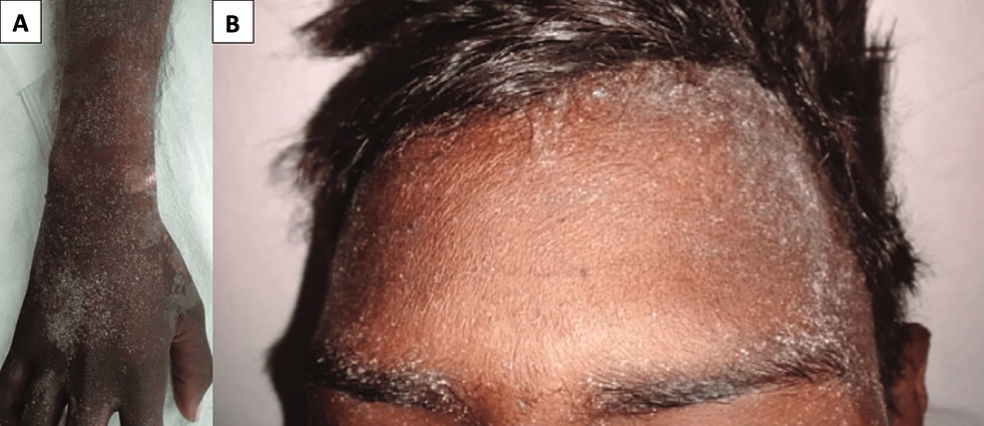
Uremic frost (A) Uremic frost on the forehead. (B) Uremic frost on the upper limb.

An example of Uremig Frost is given in Figures 5A, 5B [67].

Nail, hair, and mucosal disorders 

Nail manifestations in CKD patients are diverse, seen in about 40% of uremic patients [9]. Half-and-half nails or Lindsay's nails may be due to increased hormone-stimulating beta-melanocytes [65]. Muehrcke lines are often associated with hypoalbuminemia and conditions like cirrhosis, glomerulonephritis, nephrotic syndrome, metabolic stress, chemotherapy, trauma, and high altitude [66].

**Figure 6:**
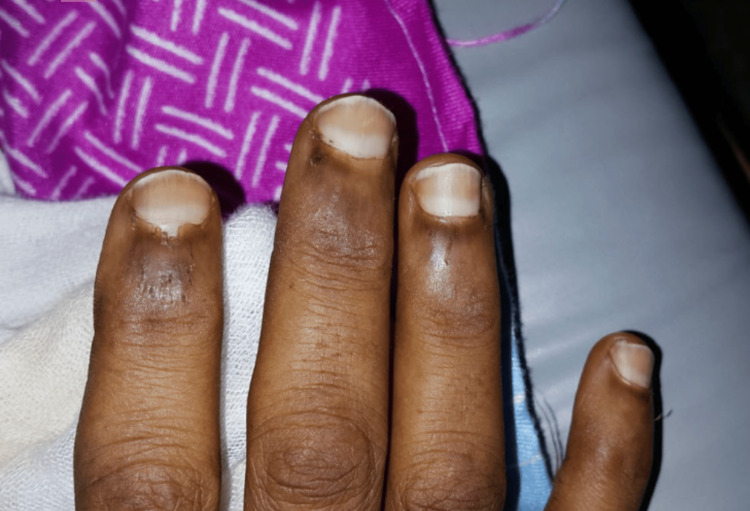
Lindsay's nails Fingernails showing typical Lindsay's nails

Lindsay's nails feature a pink-red or brown horizontal band on the distal portion, occupying nearly half of the nail length, and a dull white, ground-glass appearance in the proximal portion [9]. Mees lines (true leukonychia) have no palpable ridges and do not disappear with nail bed pressure. Muehrcke lines are twin transverse lines across the nail, visible but not palpable, and do not progress with nail growth (Figure 6) [66,68]. Other changes include splinter hemorrhages, koilonychia, onycholysis, and Beau’s lines. There is no specific treatment for these nail changes [9].

Mucosal disorders are common in HD patients, affecting more than 2% of the population [69]. CKD patients often suffer from periodontitis and inflammation of the oral mucosa, with a higher prevalence of oral lesions like taste disorders and xerostomia [69]. More than 70% of patients experience such changes, including lusterless hair [70]. Contributing factors include nutritional deficiencies, candidiasis, poor oral hygiene, and dehydration. Treatments focus on maintaining oral hygiene and addressing triggers. Hair changes in CKD patients, such as sparse scalp and body hair or diffuse alopecia, are attributed to reduced sebum production, anemia, and chronic disease stress [9].

Nonmelanoma skin cancer

CKD patients, particularly those undergoing renal replacement therapy, face a higher cancer risk compared to the general population [71]. HD patients have a 1.58-fold higher risk of developing non-melanoma skin cancer (NMSC) [72], while kidney transplant recipients have a higher prevalence of various cancers [73]. Immune suppression post-transplantation is linked to an increased risk of NMSC, non-Hodgkin lymphoma, and Kaposi sarcoma [74]. Renal transplantation is also associated with melanoma and Kaposi sarcoma [75]. The most common types of NMSC in these patients are squamous cell carcinoma (SCC) and basal cell carcinoma (BCC) [76]. The pathogenesis of skin cancer in CKD patients may involve uremic toxins and reactive oxygen species, leading to chronic systemic inflammation, genomic damage, immune dysfunction, and DNA repair impairment [77,78]. UVB Phototherapy for Uremic Pruritus has not shown an association with NMSC [79].

Immunosuppressive therapy increases the risk of skin cancer in post-transplant patients [74]. Tacrolimus-based regimens have a lower incidence of NMSC than cyclosporin, and mycophenolate is associated with a lower incidence of NMSC and squamous cell carcinoma than azathioprine [80]. Screening is crucial for diagnosing skin cancer in CKD patients; thus, dermatological examinations are recommended every six months. Diagnosis involves a visual examination by a dermatologist, who will treat lesions or refer the patient to oncology services as needed [81].

## Conclusions

CKD is a globally prevalent illness with multisystemic detrimental effects. The dermatoses observed in CKD patients are closely linked to the pro-inflammatory state often associated with this condition. In many cases, these skin signs and symptoms are indicative of underlying kidney disease or the deterioration of renal function due to elevated toxin levels. For instance, uremic toxins are linked with pruritus, and low albumin levels are associated with nail disorders and potentially undiagnosed conditions like porphyria. Despite their significance, dermatological manifestations remain under-recognized and untreated comorbidities in CKD patients. It is crucial for clinicians to thoroughly examine patients' skin, hair, and mucosal areas to diagnose and effectively treat these symptoms. Accurate interpretation of dermatological lesions can lead to effective symptomatic treatment, utilizing lotions or oral medication, thereby improving the quality of life for CKD patients. This article provides an in-depth review of existing literature on the dermatological findings in CKD patients. By offering concise and comprehensive information, it aims to enhance understanding and awareness of these comorbidities within the medical community, underscoring the importance of integrated care in managing CKD.
